# Sleep Disorders in Moyamoya Disease: Prevalence, Risk Factors, and Impact on Functional Outcomes

**DOI:** 10.2174/011570159X396815251124114647

**Published:** 2026-01-13

**Authors:** Qingbao Guo, Manli Xie, Dan Yu, Cong Han, Yuqing Gao, Qian-Nan Wang, Xiangyang Bao, Lian Duan

**Affiliations:** 1 Department of Neurosurgery, Xi'an No.9 Hospital, 151 East Section of South Second Ring Road, Xi'an, Shaanxi, 710054, China;; 2 Department of Occupational Diseases, Xi'an Central Hospital, 161 West Fifth Road, Xi'an, Shaanxi, 710003, China;; 3 Department of Neurosurgery, the Fifth Medical Centre, Chinese PLA General Hospital, 8 Dongdajie, Beijing, 100071, China;; 4 Department of Neurosurgery, the Eighth Medical Centre, Chinese PLA General Hospital, No. A17, Heishanhu Road, Beijing, 100089, China;; 5 Department of Neurosurgery, the First Medical Centre, Chinese PLA General Hospital, 28 Fuxing Road, Beijing, 100039, China

**Keywords:** Sleep disorders, moyamoya disease, underrecognized issue, MMD patients, insomnia symptoms, routine sleep screening

## Abstract

**Introduction:**

Sleep disorders are prevalent but frequently overlooked in Moyamoya Disease (MMD), with their clinical impact remaining inadequately characterized. This study aimed to determine the prevalence of sleep disorders, assess their effect on functional outcomes, and identify independent predictors in MMD patients.

**Methods:**

This prospective cohort study enrolled 320 consecutive adult MMD patients. Sleep quality, anxiety, depression, and insomnia symptoms were evaluated using the PSQI, SAS, SDS, and AIS, respectively. Two independent reviewers assessed clinical and radiological outcomes. Multivariate logistic regression was employed to identify independent risk factors for sleep disorders.

**Results:**

Of the 320 patients (184 females, mean age 45±11 years), 52.1% with sleep disorders had poor functional outcomes (mRS≥2) at admission, which persisted in 55.1% at 3 months post-EDAS. Multivariate analysis revealed that female gender (OR: 5.37, 95% CI: 2.30-12.56), older age (OR: 1.05, 95% CI: 1.01-1.09), headache (OR: 9.25, 95% CI: 1.96-43.67), alcohol consumption (OR: 3.94, 95% CI: 1.68-9.22), anxiety (OR: 4.18, 95% CI: 1.44-12.16), depression (OR: 21.22, 95% CI: 2.53-177.90), higher admission and postoperative mRS scores, and impaired hemodynamic parameters (TTP, rCBV, rCBF) were independent risk factors. Sleeping pill use was a protective factor (OR: 0.16, 95% CI: 0.06-0.42).

**Discussion and Conclusion:**

Sleep disorders are common and adversely impact functional recovery in MMD, yet remain underrecognized. The identified multivariable risk profile underscores the necessity of integrating routine sleep screening and comprehensive management into standard MMD care to improve patient prognosis.

## 1 INTRODUCTION

Moyamoya Disease (MMD) is a rare cerebrovascular disorder characterized by the steno-occlusive change in internal carotid arteries and their branches, leading to the formation of small collateral blood vessels, which resemble "puffs of smoke", termed "moyamoya" in Japanese [1]. As is well known, MMD can lead to various neurological symptoms and complications, primarily cerebral ischemia, hemorrhagic stroke, and cognitive impairment [2], which have been extensively studied. However, research regarding sleep disorders associated with MMD remains limited and underrecognized.

Sleep disorders, defined as pathological conditions involving dysregulation of sleep architecture (including duration, quality, and circadian patterns) or maladaptive sleep-related behaviors and biological functions, represent a modifiable risk factor that adversely affects both quality of life and multisystem health outcomes in affected individuals [3]. Previous studies have shown that approximately 33% of American adults report sleeping less than 7 hours per night, with 50 to 70 million people affected by sleep disorders [4, 5]. Moreover, sleep disorders have been associated with a range of neurological conditions. For example, the correlation between sleep disorders and vascular risk factors and stroke has been confirmed [6]. Sleep disorders may play a role in the occurrence and development of vascular pathology in stroke through various direct or indirect mechanisms, which has also been validated. Furthermore, the consequences of untreated sleep disorders, including cognitive dysfunction, altered mood, sleepiness, and fatigue, may hinder stroke rehabilitation, prolong hospital stays, and impact stroke outcomes and recurrence [6, 7]. Despite their prevalence, sleep disorders remain an underrecognized and undertreated issue in patients with MMD. The neglect of sleep problems in these patients may result in significant impairment in their quality of life and exacerbate the progression of MMD.

Therefore, prospective studies are designed to explore the risk factors for sleep disorders to fill this gap in the literature. By elucidating the potential link between MMD and sleep disorders, this study aims to help better understand the overall impact of sleep disorders on patients with MMD and provide information for clinical management and intervention of affected individuals.

## 2 MATERIALS AND METHODS

### 2.1 Patient Population

 This prospective analysis was conducted at the Fifth Medical Centre, Chinese PLA General Hospital, enrolling consecutive adult patients (≥18 years) diagnosed with MMD from December 2022 to May 2023. The diagnosis of MMD was confirmed using Digital Subtraction Angiography (DSA) according to the criteria established by the Research Committee on MMD (Spontaneous Occlusion of the Circle of Willis) of the Ministry of Health, Labor and Welfare, Japan, and the Japan Stroke Society's 2021 guideline [8]. Sleep disorders were diagnosed based on the International Classification of Sleep Disorders [3]. Ethical approval was obtained from the Ethics Committee of the Fifth Medical Centre, Chinese PLA General Hospital. All participants provided written informed consent prior to enrollment, in compliance with the Declaration of Helsinki. The inclusion criteria comprised: (i) Patients diagnosed with MMD using DSA; (ii) Patients diagnosed with sleep disorders based on the diagnostic criteria in the International Classification of Sleep Disorders [3]; (iii) Adult patients (age ≥ 18); (iv) Conscious and possessing basic reading, writing, and listening abilities (Montreal Cognitive Assessment (MoCA score) ≥ 18) [9]; (v) No serious diseases or complications. The exclusion criteria included: (i) Pediatric patients (age<18); (ii) Long-term use of psychotropic drugs; (iii) Patients with sleep disorders caused by tumors, chronic pain, asthma, chronic respiratory diseases, *etc*.; (iv) Patients with complex neurological comorbidities, including central nervous system (CNS) neoplasms, documented neurotrauma requiring intervention, history of intracranial surgical procedures, and confirmed neurogenetic disorders; (iv) Excessive drinking; and (v) Patients deemed unsuitable for inclusion by researchers.

During the study period, 401 patients with MMD were screened. Of these, 382 met the inclusion criteria and underwent further evaluation for sleep disorders. After excluding individuals based on exclusion criteria (n=62), a final cohort of 320 patients was enrolled in the study. Moyamoya Syndrome (MMS) was excluded from the study.

The clinical dataset is maintained at the Fifth Medical Centre, Chinese PLA General Hospital, and will be shared upon request to researchers who meet appropriate access criteria.

### 2.2 Clinical Data Collection

The study prospectively collected comprehensive clinical variables encompassing demographic characteristics (sex, age), symptom profiles, neuroimaging parameters, cerebrovascular perfusion metrics, neurological disability scores, procedure-related morbidity, and 3-month functional outcomes. Admission presentations were stratified into: (I) Ischemic manifestations comprising transient ischemic events (*e.g*., TIA, cephalalgia) and radiologically confirmed cerebral infarction; (II) Hemorrhagic events (intraventricular and intraparenchymal hemorrhage) confirmed by neuroimaging. Neurological functional status was assessed using the modified Rankin Scale (mRS) [10], a validated ordinal scale ranging from 0 (no symptoms) to 6 (fatal outcome). Baseline and final follow-up evaluations were conducted during initial hospitalization and at 3 months post-discharge, respectively. Scores ≥2 defined clinically significant disability, corresponding to the requirement for assistance in instrumental daily activities while retaining independent ambulation capacity.

### 2.3 Sleep Related Evaluation Tools

The Pittsburgh Sleep Quality Index (PSQI) [11], Self Rating Depression Scale (SDS) [12], Self Rating Anxiety Scale (SAS) [13], and Athens Insomnia Scale (AIS) [14] were conducted by well-trained neurologists. PSQI subjectively quantified sleep disturbance severity through its 21-point scale, with progressive deterioration tiers: 0-5 (good), 6-10 (fair), 11-15 (poor), 16-21 (severe). Depression and anxiety symptoms were assessed using SDS/SAS, each comprising 20 Likert-scale items (1-4 points) converted *via* standardized coefficients (raw sum×1.25), demonstrating clinical thresholds at SDS≥53 (mild:53-62, moderate:63-72, severe>72) and SAS≥50 (mild:50-59, moderate:60-69, severe>69). Sleep architecture abnormalities were further evaluated by the AIS through its 8-item construct (0-24 range), with diagnostic stratification as <4 (non-pathological), 4-6 (subclinical), >6 (insomnia diagnosis).

### 2.4 Hemodynamic Examination

Cerebrovascular perfusion dynamics were quantitatively assessed *via* Dynamic Susceptibility Contrast Magnetic Resonance Imaging (DSC-MRI) perfusion-weighted imaging on a 3T MAGNETOM Skyra platform (Siemens Healthineers) using standardized acquisition parameters and post-processing pipeline [15]. Dynamic susceptibility DSC-MRI datasets were reconstructed on a dedicated post-processing platform (Syngo.Via VB20, Siemens Healthineers) using perfusion-mapping algorithms. Manual Region-Of-Interest (ROI) placement targeted middle cerebral artery vascular territories and cerebellar cortical regions, with rigorous exclusion of infarct core regions through lesion masking protocols [16]. Cortical ROIs were defined as multiple 3–5 cm diameter circular samples along the Middle Cerebral Artery (MCA) cortical watershed zones and 1 cm diameter cerebellar cortical regions. Territorial perfusion metrics were derived from triplanar ROI averages within vascular territories. Hemispheric quantitative parameters included regional Cerebral Blood Volume (rCBV), regional Cerebral Blood Flow (rCBF), and Time-to-Peak (TTP), with rCBV/CBF ratios normalized to cerebellar reference values and TTP delays calculated as target-cerebellar differentials.

These assessments were conducted by two senior, board-certified radiologists with extensive expertise in neuroimaging and quantitative perfusion analysis. Both experts independently analyzed the data to ensure accuracy and reliability.

### 2.5 Sleep Disorders Treatment

Two experienced neurologists formulate the assessment and treatment of MMD patients with sleep disorders. The primary pharmacological agents utilized to treat sleep disorders are benzodiazepines. In cases of poor control, treatment is supplemented with melatonin agonists and orexin receptor antagonists. This stepped approach typically results in significant symptomatic improvement for the majority of patients. Given that sedatives and hypnotics are particularly associated with the risk of tolerance, dependence, and cognitive impairment, especially in long-term use, their use in certain populations (such as the elderly and children) requires careful consideration and guidance from sleep experts. Cognitive Behavioral Therapy (CBT-I) [17] for insomnia has become the gold standard non-pharmacological method for patients with poor drug treatment efficacy.

### 2.6 Operative Technique

Encephaloduroarteriosynangiosis (EDAS) was implemented bilaterally in staged procedures, excluding unilateral Moyamoya Disease (MMD) cases. Surgical prioritization targeted symptom-dominant hemispheres exhibiting greater disease burden, with contralateral interventions deferred based on hemodynamic severity. All procedures were consistently executed by a single neurosurgical team adhering to the Matsushima technique [18]. Perioperative complications were defined as adverse events occurring ≤14 days postoperatively.

### 2.7 Statistical Analyses

Statistical analyses were conducted using appropriate methods for variable types. Continuous variables were compared using Welch's t-test, while categorical variables were assessed through Pearson's χ² test, supplemented by Fisher's exact test when expected cell counts were small (*e.g*., <5). Correlation analyses employed Spearman's rank correlation coefficient to assess associations between continuous and ordinal variables. To evaluate potential risk factors for sleep disorders, a multivariable logistic regression framework was constructed. The model incorporated baseline clinical and imaging variables that demonstrated preliminary associations in univariate analyses (*P<*0.05). To account for potential confounding, the final regression model included variables adjusted for age, sex, education level, comorbidities, and other relevant covariates identified a priori as potential confounders based on their known associations with sleep disorders. Non-linear associations between continuous predictors and sleep disturbance outcomes were investigated using Restricted Cubic Spline (RCS) transformations within the logistic regression framework. The RCS Logit model was implemented to flexibly model non-linear relationships, with knot placement determined through systematic comparison of Akaike Information Criterion (AIC) values. Ultimately, a 7-knot configuration was selected, positioned at the 2^nd^, 18^th^, 34^th^, 50^th^, 66^th^, 82^nd^, and 98^th^ percentiles of the predictor distribution. Unadjusted estimates from univariate analyses were reported alongside confounder-adjusted estimates (odds ratios with 95% confidence intervals) from the final multivariable model. The precision of estimates was quantified using 95% confidence intervals to assess statistical significance and the robustness of associations. All analyses were performed using R statistical software (version 4.2.2, R Foundation) and MedCalc (version 20.2.18, MedCalc Software Ltd), with graphical representations generated through specialized packages in both platforms.

## 3 RESULTS

### 3.1 Sample Size

A sample size of 236 patients was calculated to yield a 95% confidence interval estimate with a precision of ±6%, assuming a sample proportion of 33% [3]. This interval represents the range within which the true population proportion is estimated to lie. Anticipating a potential dropout rate of 5%, the total required sample size was adjusted upward to 249. Our study ultimately enrolled 320 participants, which exceeded the calculated requirements to ensure adequate statistical power.

### 3.2 Patient Characteristics

The baseline characteristics table (Table **1**) presents key demographic and clinical variables related to sleep disorders in the study population. The study analyzed 320 participants, with 129 diagnosed with sleep disorders and 191 without. Participants with sleep disorders were, on average, older (48±9 years) than those without (43±11 years). TTP delay was longer in the sleep disorder group (2.12±1.58 s) than in the non-sleep disorder group (1.44±1.13 s). The relative rCBV and rCBF ratios were lower in participants with sleep disorders (0.92±0.42 and 1.08±0.32) compared to those without (1.28±0.54 and 1.24±0.65). The gender distribution demonstrated a higher female predominance in the sleep disorder cohort (36.4% male *vs.* 63.6% female) compared to the non-sleep disorder group (46.6% male *vs.* 53.4% female). The most common initial symptom was cerebral hemorrhage for both groups, followed by TIA. Insomnia disorder was predominant among those with sleep disorders, while a high percentage had no sleep disorder. Comorbid conditions like diabetes, hyperlipidemia, and hyperhomocysteinemia were slightly higher in the sleep disorder group. Suzuki Stage 4 was most common in both groups, but less prevalent in the sleep disorder group. Different lifestyle factors and medication use varied between the two groups, with anxiety, depression, and poor sleep quality higher among those with sleep disorders. In the AIS assessment, insomnia was more frequently reported in the sleep disorder group. Postoperative complications and mRS scores showed worse outcomes in participants with sleep disorders.

### 3.3 Association Between MMD Sleep Disorders and Functional Outcome

**Fig. (1):**
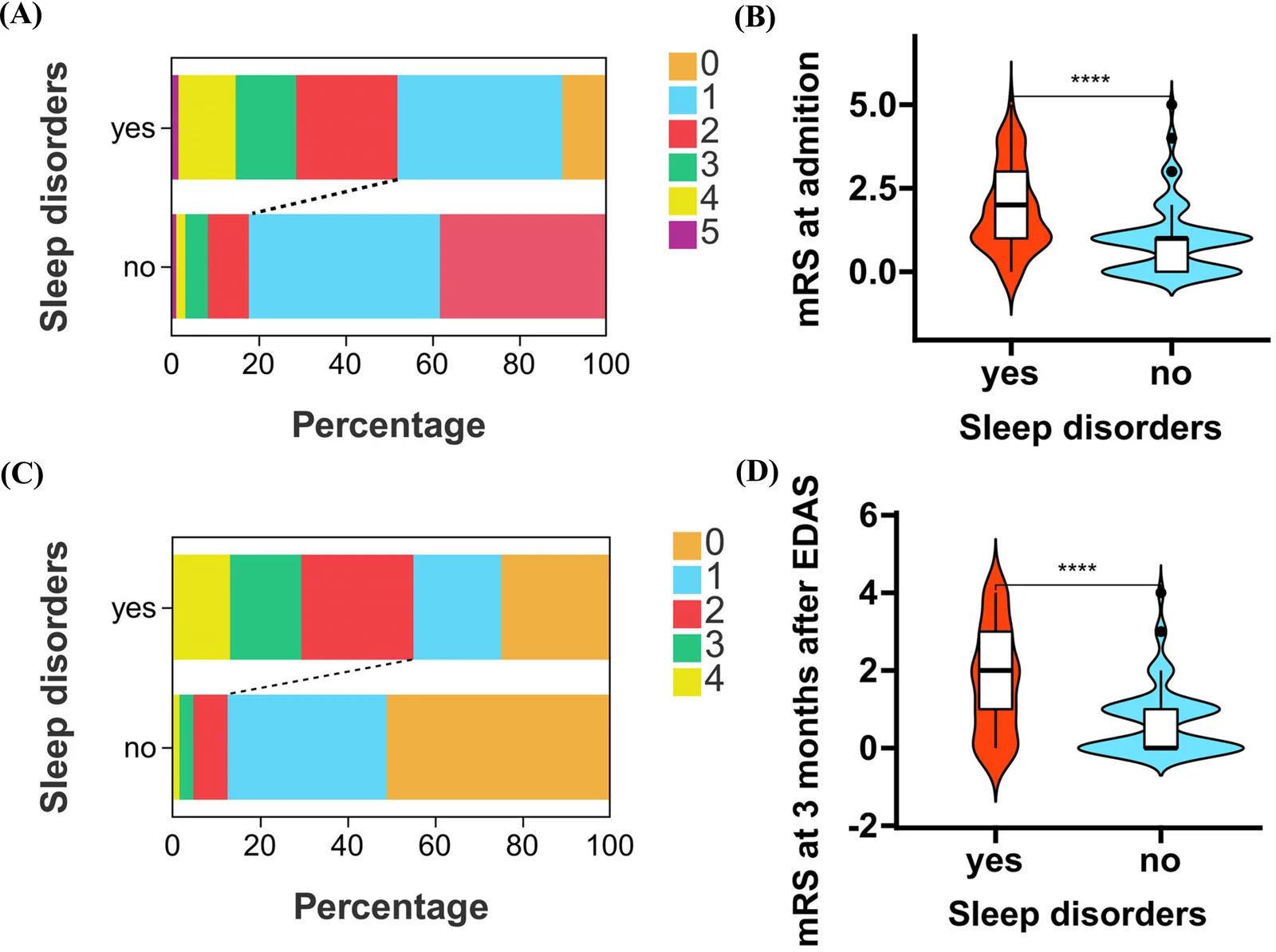
Association between sleep disorders and functional outcomes in MMD patients undergoing EDAS. (**A**) Distribution of mRS at admission stratified by sleep disorder status (percentage cohort representation). (**B**) Comparative analysis of admission mRS scores between groups. (**C**) Postoperative mRS score distribution at 3-month follow-up (percentage cohort representation). (**D**) Longitudinal comparison of mRS scores post-EDAS.

Among MMD patients with sleep disorders, 52.1% (67/129) experienced poor functional outcome (mRS≥2). Sleep disorder patients exhibited higher mRS scores at admission compared to MMD patients without sleep disorders (*P <*0.001, Fig. **1A**, **B**). The analysis of mRS scores at 3 months post-EDAS revealed similar results, with 55.1% (71/129) showing a poor functional outcome (mRS≥2). Once again, individuals with sleep disorders had higher mRS scores than those without (*P<*0.001; Fig. **1C**, **D**). These results suggest that sleep disturbance significantly affects the prognosis of patients with MMD. Therefore, exploring the influencing factors of sleep disorders and standardizing the management and monitoring of sleep disorders is of great significance to improve the prognosis of patients.

### 3.4 Univariate Logistic Regression Analysis

 Table **2** presents the associations between clinical characteristics and sleep disorders. Females exhibited a trend towards a higher risk of the event (OR: 1.52, 95% CI: 0.96–2.41, *p* = 0.072). Age was significantly associated with event occurrence, with each additional year increasing the odds by 4% (OR: 1.04, 95% CI: 1.02–1.07, *p <* 0.001). Initial symptoms of headache were also significantly associated with a higher risk of the event (OR: 3.25, 95% CI: 1.13–9.38, *p* = 0.029). Alcohol consumption markedly increased the odds of the event (OR: 3.03, 95% CI: 1.88–4.87, *p <* 0.001). Mild anxiety presented a significant association (OR: 2.18, 95% CI: 1.15–4.14, *p =* 0.017), alongside moderate anxiety (OR: 3.15, 95% CI: 1.02–9.66, *p* = 0.045). Mild depression was associated with a significant increase in event odds (OR: 3.99, 95% CI: 2.02–7.89, *p <* 0.001). Postoperative TIA complications significantly correlated with the event (OR: 4.35, 95% CI: 1.13–16.74, *p =* 0.033). Higher mRS scores at admission (mRS 1: OR: 3.28, *p <* 0.001; mRS 2: OR: 9.36, *p <* 0.001; mRS 3: OR: 10.11, *p <* 0.001; mRS 4: OR: 23.87, *p <* 0.001) and at three months post-EDAS (mRS 2: OR: 6.67, *p <* 0.001; mRS 3: OR: 10.61, *p <* 0.001; mRS 4: OR: 17.18, *p <* 0.001) indicated strong predictive value regarding the event occurrence. Delay in Time-to-Peak (TTP) significantly increased odds of event occurrence (OR: 1.47, 95% CI: 1.22–1.76, *p <* 0.001), as did reductions in relative cerebral blood volume (rCBV) (OR: 0.19, 95% CI: 0.11–0.33, *p <* 0.001) and relative cerebral blood flow (rCBF) (OR: 0.54, 95% CI: 0.34–0.86, *p =* 0.009).

In summary, significant associations were found for age, initial symptoms (specifically headache), alcohol consumption, anxiety and depression levels, postoperative complications, mRS scores, TTP delay, rCBV, and rCBF ratios with respect to event occurrence, while no significant associations were noted for sex, diabetes, hyperlipidemia, hyperhomocysteinemia, smoking status, and various other initial symptoms. (Table **2**).

### 3.5 Multivariate Logistic Regression Analysis

 All variables with a significance level of *P<*0.1 in the univariate analysis were included in the multivariate logistic regression model. In multivariate logistic regression analysis, significant associations with sleep disorders were identified. Females had 5.37 times the odds compared to males (95% CI: 2.30-12.56, *p<*0.001). Age was a risk factor, with each additional year increasing the odds by 1.05 (95% CI: 1.01-1.09, *p=*0.013). Headaches were highly predictive of disorders (OR: 9.25, 95% CI: 1.96-43.67, *p=*0.005). Lifestyle factors like alcohol consumption increased the odds by nearly four times (OR: 3.94, 95% CI: 1.68-9.22, *p=*0.002), while sleeping pill use was protective (OR: 0.16, 95% CI: 0.06-0.42, *p<*0.001). Anxiety and depression were strong predictors, with mild anxiety (OR: 4.18, 95% CI: 1.44-12.16, *p=*0.009) and moderate depression (OR: 21.22, 95% CI: 2.53-177.90, *p=*0.005) significantly increasing the likelihood of sleep disorders. The mRS score at admission, was a significant predictor, particularly scores of 2 at admission (OR: 13.22, 95% CI: 3.06-57.21, *p<*0.001) and scores of 3 or 4 at 3 months after EDAS (OR: 13.86, 95% CI: 2.42-79.44, *p=*0.003; OR: 60.63, 95% CI: 6.22-591.18, *p<*0.001, respectively). Radiological evaluation, like TTP delay (OR: 1.79, 95% CI: 1.26-2.55, *p=*0.001) and reductions in rCBV and rCBF ratios (OR: 0.19, 95% CI: 0.07-0.51, *p=*0.001; OR: 0.44, 95% CI: 0.20-0.96, *p=*0.040, respectively) also had significant correlations (Table **3**).

### 3.6 Restricted Cubic Splines (RCS)

**Fig. (2):**
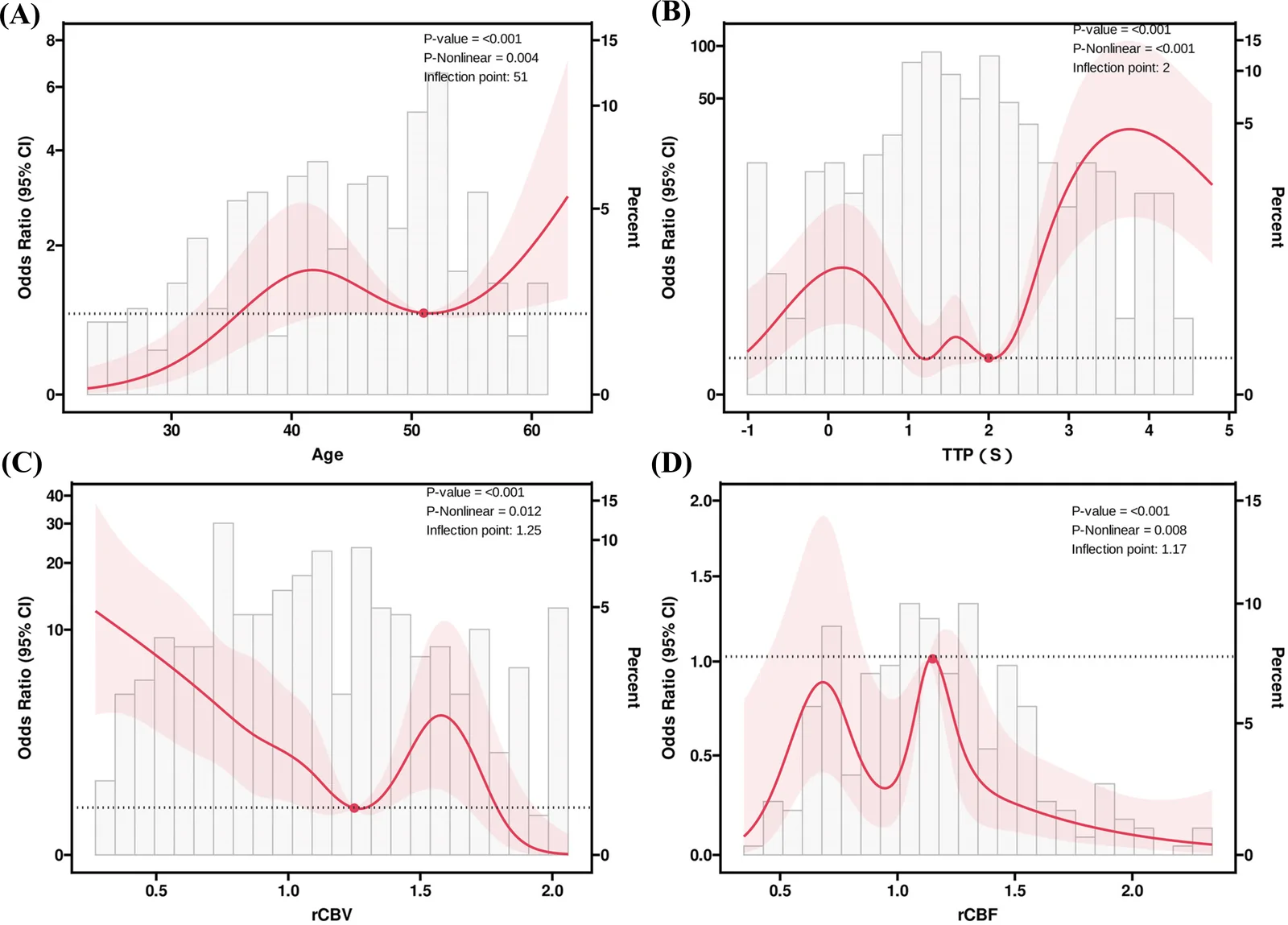
The RCS analysis of nonlinear relationships between physiological parameters and sleep disorder risk. (**A**) Age (years); (**B**) TTP delay (seconds); (**C**) rCBV (ratio); (**D**) rCBF (ratio).

 The RCS analysis indicated a non-linear association between age, TTP delay, rCBV, and rCBF with sleep disorders. Restricted cubic spline analysis revealed nonlinear associations between key predictors and sleep disorder risk, with inflection points identified at age=51 years (Fig. **2A**), time-to-peak (TTP)=2 s (Fig. **2B**), relative cerebral blood volume (rCBV)=1.25 (Fig. **2C**), and relative cerebral blood flow (rCBF)=1.17 (Fig. **2D**). These critical thresholds demarcated distinct phases in parameter-outcome relationships, indicating potential transition points where the direction or magnitude of association between physiological measures and sleep disturbance risk underwent fundamental alterations.

## 4 DISCUSSION

To the best of our knowledge, this study represents the first investigation into the impact of sleep disorders on MMD. Our findings revealed that 40.3% (129/320) of MMD patients had sleep disorders, yet only 15.5% (20/129) of them were effectively managed with medication, suggesting a common occurrence of untreated sleep disorders in MMD. Subsequently, we observed a significant impact of sleep disorders on patient outcomes. Finally, we investigated the factors influencing sleep disorders and identified females, age, headaches, alcohol consumption, sleeping pill usage, anxiety, depression, the mRS score at admission, mRS at 3 months post-EDAS, and radiological evaluation parameters, including TTP, rCBV, and rCBF as independent risk factors.

In the general population, chronic sleep disorders have been associated with increased risks of cardiovascular diseases [19], metabolic disorders [20], cognitive impairment, and mental health conditions [21]. Furthermore, in surgical patients, sleep disorders have been shown to negatively impact postoperative recovery, including delayed wound healing, prolonged hospitalization, and higher rates of postoperative complications [4, 22]. However, the impact of sleep disorders on MMD has only recently come to attention. While there is limited data directly linking the effects of the consequences of altered sleep patterns to increased patient morbidity and mortality, the consequences of inadequate sleep may impact inpatient stroke rehabilitation endeavors. Regardless of their specific cause, poor sleep quality or duration substantially hinders a patient's rehabilitation for numerous reasons. Sleep disorders obstruct the restorative processes that take place during uninterrupted sleep [23]. Stenotic involvement of both the Internal Carotid Arteries (ICA) and Posterior Cerebral Arteries (PCA) may disrupt global cerebral perfusion dynamics in MMD, while the characteristic formation of collateral moyamoya vasculature may reflect underlying hemodynamic compromise associated with diminished cerebrovascular reserve capacity and impaired autoregulatory mechanisms [24]. Therefore, patients with MMD are more susceptible to the effects of sleep disorders. Our study revealed that MMD patients with sleep disorders exhibit poorer functional outcomes, aligning with findings in cardiovascular disease [25]. Interestingly, both patients who experienced postoperative cerebral hemorrhage had preoperative sleep disorders. Further large-scale studies are needed to confirm whether preoperative sleep disorders increase the risk of postoperative cerebral hemorrhage.

Our study found that females were an independent risk factor for sleep disorders in MMD patients. Although this is the first finding in MMD patients, sleep disorders in women are widely studied in other disorders. Women’s responses to sleep disorders, deprivation, and deficiency lead to specific health outcomes. Biological changes during menstruation, pregnancy, and menopause highlight the need to study women’s sleep patterns. Each life stage, from childhood to menopause, presents unique challenges in managing sleep disturbances. Recent research highlights the vital role of sex hormones in regulating sleep and arousals in women. Hormonal fluctuations increase the risk of sleep disorders, such as poor sleep quality and conditions like Obstructive Sleep Apnea (OSA), restless legs syndrome, and insomnia [26]. These findings align with our results, emphasizing the significance of addressing sleep disorders in women with MMD.

Our study also found that age significantly affects sleep, which is consistent with previous studies. Existing research demonstrates age-associated alterations in sleep architecture, as evidenced by both subjective reports and objective assessments, with elderly females exhibiting a higher prevalence of sleep disturbances. Characteristic changes include circadian rhythm shifts manifesting as advanced sleep phase onset, sleep fragmentation with reduced continuity, diminished total sleep duration, and clinically significant daytime somnolence, all objectively quantifiable through polysomnographic measures [27, 28]. By using objective measurement methods such as Polysomnography (PSG), research has supported the descriptions of sleep disturbances in subjective reports. A comprehensive meta-analysis of 65 polysomnographic studies involving 3,577 participants spanning the adult lifespan demonstrated progressive age-related modifications in sleep architecture, characterized by increased proportional duration of light sleep stages (N1/N2), accompanied by corresponding reductions in both Rapid Eye Movement (REM) sleep and Slow Wave Sleep (SWS) components [29]. Interestingly, we found that the age inflection point was 51, which coincides with the age of menopause in women.

Headache is common in MMD; however, the effect of headache on sleep disorders in MMD patients has never been considered before. Our study found that headache significantly affected sleep disturbance in MMD patients. Headaches and sleep disorders are intricately linked. Both conditions are common in the general population, but their relationship extends beyond coincidence. Sleep disorders may worsen headaches, and vice versa. Treating sleep disorders can also positively impact headaches [30]. In patients with MMD, determining whether headaches stem from the disease itself or from concomitant sleep disorders is crucial, as it influences the choice of tailored treatment. Therefore, we recommend that patients with MMD headaches be screened for sleep disorders.

Another significant concern pertains to the independent role of alcohol consumption as a risk factor for sleep disorders in patients with MMD. This is an interesting finding, as previous studies have found that alcohol consumption acts as a sedative, reducing the latency to sleep [31] and, therefore, can be actively used to alleviate insomnia [32], which is inconsistent with our findings. However, recent studies indicate a correlation between aging, increased alcohol consumption in the elderly, and the heightened prevalence of insomnia [28]. Therefore, this age group warrants specific attention. Patients with Meniere’s disease who are above the inflection point age (≥51) should practice total abstinence from alcohol.

In our study, only 15.5% of patients with MMD had sleep disorders that were controlled by medication, indicating that this issue is not taken seriously by MMD patients and physicians. Prior research indicates that utilizing opioid analgesics or sedatives can enhance sleep quality, leading to improved survival rates among cancer patients [33]. For individuals suffering from chronic insomnia, regardless of the underlying causes, the highest recommendation by the American Academy of Sleep Medicine is behavioral intervention therapy [34]. Therefore, we suggest that MMD patients with sleep disorders should pay attention to the treatment of sleep disorders while actively treating the primary disease.

This study has provided novel insights into the psychological implications of sleep disorders in patients with MMD. The observed prevalence of anxiety and depression among MMD patients with sleep disorders is notably higher than in those without sleep disorders. Although the risk of increased anxiety and depression associated with MMD-related sleep disorders has never been studied or taken seriously before, sleep disorders have been recognized in the broader literature as a comorbidity that can exacerbate psychological distress. Prior studies have shown that disturbed sleep can precipitate or worsen anxiety and depressive symptoms, highlighting a bidirectional relationship between sleep and mental health [35]. In the context of MMD, a chronic and progressive cerebrovascular disease, the physical symptoms and the associated psychosocial stress could further complicate this interplay. Given the complex, multidimensional nature of MMD, the integration of mental health evaluation into routine care is essential. Our findings align with those of Jacobsen *et al*., who emphasize the need for early identification and treatment of mental health issues in patients with chronic illnesses to improve overall prognosis [36]. Future research should orient towards longitudinal studies to track the progression and interaction of sleep disorders with anxiety and depression over time in the MMD population, further informing targeted treatment strategies that encompass the multidisciplinary management of the disease.

The hemodynamic patterns in MMD are highly complex. The disease is characterized not only by steno-occlusive change in the Internal Carotid Artery (ICA) but also by similar pathological changes in the Posterior Cerebral Artery (PCA) [37]. Compensatory cerebrovascular networks emerge in MMD, comprising posterior circulation-derived leptomeningeal anastomoses, external carotid artery-connected transdural collateral pathways, and characteristic moyamoya vessels, collectively mediating revascularization of cerebral territories affected by chronic hypoperfusion [38]. However, surprisingly, Yamada and colleagues elucidated that changes in the PCA significantly influence the cerebral hemodynamics of patients with MMD, whereas alterations in the ICA do not show a similar correlation [39]. These results have confirmed that impaired hemodynamics are common in patients with MMD. Although there have been no relevant literature reports on the impact of sleep disorders on the hemodynamics of patients with MMD, research on the effects of sleep disorders on cerebral hemodynamics has been well documented. Accumulating evidence suggests marked disruptions in cerebral perfusion among patients with Obstructive Sleep Apnea (OSA). Studies have reported reduced Cerebral Blood Flow (CBF) [18], along with fluctuating elevations in both Blood Pressure (BP) and Cerebral Blood Flow Velocity (CBFV) [40]. The findings of our study reveal a notable association between sleep disorders and perfusion parameters in patients with MMD. Specifically, our research demonstrated that more severe TTP delay, poorer rCBV, and rCBF were linked to the presence of sleep disorders in MMD patients compared to those without sleep disorders. These findings collectively suggest that sleep disturbances may contribute to the progression or clinical manifestation of cerebrovascular hypoperfusion in MMD patients. The significant correlations between hemodynamic parameters and sleep-related pathology highlight the necessity of incorporating cerebrovascular reserve evaluation into comprehensive clinical strategies for managing sleep disorders within this population.

Previous studies have shown that under various medical conditions, sleep disorders can hinder the recovery process and exacerbate physical disabilities [41], which is consistent with our research findings. In contrast, the more rapid functional improvement observed in MMD patients without sleep disorders post-surgery underscores the positive influence of good sleep quality on rehabilitation outcomes. These results are supported by the work of Smith *et al*. [42], who found a similar correlation between sleep quality and post-surgical recovery in a broader medical context. The slower postoperative recovery trajectory in MMD patients with sleep disorders may necessitate more intensive or prolonged rehabilitation efforts. As indicated in a study by Krieger *et al*. [43], tailored rehabilitation programs that incorporate sleep management may be critical in optimizing functional recovery in MMD.

The correlation between sleep disorders and treatment outcomes in patients with MMD revealed that patients with sleep disorders showed quicker functional recovery postoperatively after receiving standardized treatment, as evidenced by a significant reduction in mRS scores within 3 months of surgery. Such findings signify the potential benefits of a sleeping pill treatment, especially in the context of sleep-related comorbidities. Contrastingly, the group of MMD patients with sleep disorders that did not follow a standardized treatment regimen experienced a less favorable postoperative recovery trajectory. The importance of incorporating sleep evaluation and management into the treatment protocol for MMD patients is echoed in the literature. Research by Hasan *et al*. [44] discusses how sleep disorders can affect neuroplasticity and recovery potential after brain injury. Thus, holistic treatment models that address sleep may provide a distinct recovery advantage. These observations advocate for a more integrative approach to treatment and suggest that ignoring sleep disorders in MMD patients could impede postoperative recovery. As recommended by Culebras *et al*. [45], therapeutic strategies that simultaneously address cerebrovascular pathology and sleep disturbances may optimize patient outcomes. Future investigations should aim to study larger cohorts over extended periods to further understand how standardized sleep disorder management impacts long-term recovery in MMD. This holistic view could establish more refined and specific guidelines for MMD treatment protocols.

## LIMITATIONS

 This investigation has several noteworthy limitations that warrant critical evaluation. Firstly, the exclusive recruitment of participants from a single tertiary center raises concerns regarding geographic and ethnic homogeneity, potentially introducing selection bias that may limit population generalizability. Secondly, diagnostic classification of sleep disorders depended on clinician-administered assessment scales rather than objective neurophysiological monitoring through Polysomnography (PSG) or multichannel Electroencephalography (EEG), a methodological constraint that may affect phenotypic precision. Thirdly, the semiquantitative characterization of cerebral hemodynamics *via* perfusion MRI provides relative rather than absolute perfusion metrics, necessitating technical standardization to improve inter-study comparability and clinical interpretability.

## CONCLUSION

Sleep disorders are common and adversely impact functional recovery in MMD, yet remain underrecognized. The identified multivariable risk profile underscores the necessity of integrating routine sleep screening and comprehensive management into standard MMD care to improve patient prognosis.

## Figures and Tables

**Table 1:** Patient demographics and baseline characteristics.

**Characteristic**	**Sleep Disorders**		
**Overall, N = 320** **^1^**	**Yes, N = 129** **^1^**	**No, N = 191** **^1^**
**Age**	45 ± 11	48 ± 9	43 ± 11
**BMI**	23.10 ± 2.81	23.12 ± 2.70	23.09 ± 2.89
**TTP delay** **, s**	1.72 ± 1.37	2.12 ± 1.58	1.44 ± 1.13
**rCBV ratio, %**	1.14 ± 0.53	0.92 ± 0.42	1.28 ± 0.54
**rCBF ratio, %**	1.18 ± 0.55	1.08 ± 0.32	1.24 ± 0.65
**Sex**			
Male	136 (42.5%)	47 (36.4%)	89 (46.6%)
Female	184 (57.5%)	82 (63.6%)	102 (53.4%)
**Diagnosis**			
MMD	299 (93.4%)	120 (93.0%)	179 (93.7%)
MMS	21 (6.6%)	9 (7.0%)	12 (6.3%)
**Initial Symptom**			
TIA	85 (26.6%)	34 (26.4%)	51 (26.7%)
Headache	19 (5.9%)	13 (10.1%)	6 (3.1%)
Cerebral hemorrhage	188 (58.8%)	73 (56.6%)	115 (60.2%)
Epilepsy	7 (2.2%)	3 (2.3%)	4 (2.1%)
Cerebral infarction	15 (4.7%)	4 (3.1%)	11 (5.8%)
Other	6 (1.9%)	2 (1.6%)	4 (2.1%)
**Laterality**			
Bilateral	164 (51.3%)	71 (55.0%)	93 (48.7%)
Unilateral	156 (48.8%)	58 (45.0%)	98 (51.3%)
**Types of Sleep Disorders**			
No	203 (63.4%)	29 (22.5%)	174 (91.1%)
Insomnia disorder	112 (35.0%)	96 (74.4%)	16 (8.4%)
Sleep-related breathing disorders	2 (0.6%)	1 (0.8%)	1 (0.5%)
**Diabetes**			
No	228 (71.3%)	92 (71.3%)	136 (71.2%)
Yes	92 (28.8%)	37 (28.7%)	55 (28.8%)
**Hyperlipidemia**			
Yes	142 (44.4%)	62 (48.1%)	80 (41.9%)
No	178 (55.6%)	67 (51.9%)	111 (58.1%)
**Hyperhomocysteinemia**			
No	237 (74.1%)	98 (76.0%)	139 (72.8%)
Yes	83 (25.9%)	31 (24.0%)	52 (27.2%)
**Suzuki Stage**			
1	1 (0.3%)	1 (0.8%)	0 (0.0%)
2	5 (1.6%)	3 (2.3%)	2 (1.0%)
3	9 (2.8%)	2 (1.6%)	7 (3.7%)
4	154 (48.1%)	54 (41.9%)	100 (52.4%)
5	119 (37.2%)	54 (41.9%)	65 (34.0%)
6	32 (10.0%)	15 (11.6%)	17 (8.9%)
**Smoking**			
No	274 (85.6%)	115 (89.1%)	159 (83.2%)
Yes	46 (14.4%)	14 (10.9%)	32 (16.8%)
**Alcohol**			
No	207 (64.7%)	64 (49.6%)	143 (74.9%)
Yes	113 (35.3%)	65 (50.4%)	48 (25.1%)
**Sleeping Pill**			
No	242 (75.6%)	109 (84.5%)	133 (69.6%)
Yes	78 (24.4%)	20 (15.5%)	58 (30.4%)
**SAS**			
No	261 (81.6%)	95 (73.6%)	166 (86.9%)
Mild anxiety	45 (14.1%)	25 (19.4%)	20 (10.5%)
Moderate anxiety	14 (4.4%)	9 (7.0%)	5 (2.6%)
**SDS**			
No	269 (84.1%)	94 (72.9%)	175 (91.6%)
Mild depression	44 (13.8%)	30 (23.3%)	14 (7.3%)
Moderate depression	7 (2.2%)	5 (3.9%)	2 (1.0%)
**PSQI**			
Fair sleep	77 (24.1%)	17 (13.2%)	60 (31.4%)
Moderate sleep	139 (43.4%)	26 (20.2%)	113 (59.2%)
Poor sleep	104 (32.5%)	86 (66.7%)	18 (9.4%)
**AIS**			
Insomnia	104 (32.5%)	87 (67.4%)	17 (8.9%)
No	59 (18.4%)	11 (8.5%)	48 (25.1%)
Possible insomnia	157 (49.1%)	31 (24.0%)	126 (66.0%)
**Postoperative Complications**			
No	284 (88.8%)	108 (83.7%)	176 (92.1%)
TIA	11 (3.4%)	8 (6.2%)	3 (1.6%)
Cerebral infarction	15 (4.7%)	6 (4.7%)	9 (4.7%)
Visual impairment	1 (0.3%)	1 (0.8%)	0 (0.0%)
Fever	3 (0.9%)	2 (1.6%)	1 (0.5%)
Speech disorder	3 (0.9%)	1 (0.8%)	2 (1.0%)
Scalp infection	1 (0.3%)	1 (0.8%)	0 (0.0%)
Cerebral hemorrhage	2 (0.6%)	2 (1.6%)	0 (0.0%)
**mRS at Admission**			
0	86 (26.9%)	13 (10.1%)	73 (38.2%)
1	133 (41.6%)	49 (38.0%)	84 (44.0%)
2	48 (15.0%)	30 (23.3%)	18 (9.4%)
3	28 (8.8%)	18 (14.0%)	10 (5.2%)
4	21 (6.6%)	17 (13.2%)	4 (2.1%)
5	4 (1.3%)	2 (1.6%)	2 (1.0%)
**mRS at 3 Months after EDAS**			
0	129 (40.4%)	32 (24.8%)	97 (51.1%)
1	95 (29.8%)	26 (20.2%)	69 (36.3%)
2	48 (15.0%)	33 (25.6%)	15 (7.9%)
3	27 (8.5%)	21 (16.3%)	6 (3.2%)
4	20 (6.3%)	17 (13.2%)	3 (1.6%)

**Note:** ^1^Mean ± SD; n (%).

**Table 2:** Univariate associations between clinical variables and sleep disorder risk.

**Characteristic**	**N**	**Event N**	**OR** ^1^	**95% CI** ^1^	***p*** **-value** ^2^
**Sex**					
Male	136	47	—	—	
Female	184	82	1.52	0.96, 2.41	0.072
**Age**	320	129	1.04	1.02, 1.07	<0.001***
**Initial ** **Symptom**					
TIA	85	34	—	—	
Headache	19	13	3.25	1.13, 9.38	0.029*
Cerebral hemorrhage	188	73	0.95	0.56, 1.61	0.854
Epilepsy	7	3	1.13	0.24, 5.35	0.882
Cerebral infarction	15	4	0.55	0.16, 1.85	0.332
Other	6	2	0.75	0.13, 4.32	0.748
**Laterality**					
Bilateral	164	71	—	—	
Unilateral	156	58	0.78	0.50, 1.21	0.265
**Diabetes**					
No	228	92	—	—	
Yes	92	37	0.99	0.61, 1.63	0.982
**Hyperlipidemia**					
Yes	142	62	—	—	
No	178	67	0.78	0.50, 1.22	0.276
**Hyperhomocysteinemia**					
No	237	98	—	—	
Yes	83	31	0.85	0.51, 1.41	0.523
**Suzuki Stage**					
1	1	1	—	—	
2	5	3	0.00	0.00, Inf	0.987
3	9	2	0.00	0.00, Inf	0.986
4	154	54	0.00	0.00, Inf	0.986
5	119	54	0.00	0.00, Inf	0.987
6	32	15	0.00	0.00, Inf	0.987
**Smoking**					
No	274	115	—	—	
Yes	46	14	0.60	0.31, 1.18	0.143
**Alcohol**					
No	207	64	—	—	
Yes	113	65	3.03	1.88, 4.87	<0.001***
**Sleeping Pill**					
No	242	109	—	—	
Yes	78	20	0.42	0.24, 0.74	0.003**
**BMI**	320	129	1.00	0.93, 1.09	0.915
**SAS**					
No	261	95	—	—	
Mild anxiety	45	25	2.18	1.15, 4.14	0.017*
Moderate anxiety	14	9	3.15	1.02, 9.66	0.045*
**SDS**					
No	269	94	—	—	
Mild depression	44	30	3.99	2.02, 7.89	<0.001***
Moderate depression	7	5	4.65	0.89, 24.45	0.069
**Postoperative Complications**					
No	284	108	—	—	
TIA	11	8	4.35	1.13, 16.74	0.033*
Cerebral infarction	15	6	1.09	0.38, 3.14	0.878
Visual impairment	1	1	9.38 ×10^06	0.00, Inf	0.991
Fever	3	2	3.26	0.29, 36.37	0.337
Speech disorder	3	1	0.81	0.07, 9.09	0.868
Scalp infection	1	1	9.38 ×10^06	0.00, Inf	0.991
Cerebral hemorrhage	2	2	9.38 ×10^06	0.00, Inf	0.988
**mRS at Admission**					
0	86	13	—	—	
1	133	49	3.28	1.65, 6.51	<0.001***
2	48	30	9.36	4.08, 21.47	<0.001***
3	28	18	10.11	3.82, 26.73	<0.001***
4	21	17	23.87	6.92, 82.36	<0.001***
5	4	2	5.62	0.73, 43.48	0.098
**mRS at 3 Months after EDAS**					
0	129	32	—	—	
1	95	26	1.14	0.63, 2.09	0.665
2	48	33	6.67	3.22, 13.83	<0.001***
3	27	21	10.61	3.94, 28.59	<0.001***
4	20	17	17.18	4.72, 62.45	<0.001***
**TTP delay, s**	320	129	1.47	1.22, 1.76	<0.001***
**rCBV ratio, %**	320	129	0.19	0.11, 0.33	<0.001***
**rCBF ratio, %**	320	129	0.54	0.34, 0.86	0.009**

**Note:** ^1^OR = Odds Ratio, CI = Confidence Interval. ^2^*p<0.05; **p<0.01; ***p<0.001.

**Table 3:** Independent predictors of sleep disorders identified through multivariable analysis.

**Characteristic**	**N**	**Event N**	**OR** ^1^	**95% CI** ^1^	***p*** **-value** ^2^
**Sex**					
Male	135	47	—	—	
Female	184	82	5.37	2.30, 12.56	<0.001***
**Age**	319	129	1.05	1.01, 1.09	0.013*
**Initial symptom**					
TIA	84	34	—	—	
Headache	19	13	9.25	1.96, 43.67	0.005**
Cerebral hemorrhage	188	73	1.08	0.43, 2.72	0.871
Epilepsy	7	3	0.52	0.04, 7.50	0.629
Cerebral infarction	15	4	1.05	0.15, 7.31	0.962
Other	6	2	0.15	0.01, 2.40	0.179
**Alcohol**					
No	206	64	—	—	
Yes	113	65	3.94	1.68, 9.22	0.002**
**Sleeping Pill**					
No	241	109	—	—	
Yes	78	20	0.16	0.06, 0.42	<0.001***
**SAS**					
No	260	95	—	—	
Mild anxiety	45	25	4.18	1.44, 12.16	0.009**
Moderate anxiety	14	9	14.57	2.45, 86.75	0.003**
**SDS**					
No	268	94	—	—	
Mild depression	44	30	6.69	2.18, 20.48	<0.001***
Moderate depression	7	5	21.22	2.53, 177.90	0.005**
**Postoperative Complications**					
No	284	108	—	—	
TIA	11	8	1.30	0.14, 12.14	0.816
Cerebral infarction	14	6	2.88	0.59, 14.03	0.192
Visual impairment	1	1	1.98 ×10^05	0.00, Inf	0.996
Fever	3	2	5.48	0.19, 158.03	0.322
Speech disorder	3	1	0.86	0.03, 28.32	0.935
Scalp infection	1	1	98,103.85	0.00, Inf	0.996
Cerebral hemorrhage	2	2	2.78 ×10^07	0.00, Inf	0.992
**mRS at Admission**					
0	85	13	—	—	
1	133	49	3.77	1.18, 12.10	0.026*
2	48	30	13.22	3.06, 57.21	<0.001***
3	28	18	7.06	1.46, 34.21	0.015*
4	21	17	12.66	1.79, 89.41	0.011*
5	4	2	1.04	0.05, 21.15	0.980
**mRS at 3 Months after EDAS**					
0	129	32	—	—	—
1	95	26	0.56	0.21, 1.50	0.249
2	48	33	3.35	0.97, 11.50	0.055
3	27	21	13.86	2.42, 79.44	0.003**
4	20	17	60.63	6.22, 591.18	<0.001***
**TTP delay, s**	319	129	1.79	1.26, 2.55	0.001**
**rCBV ratio, %**	319	129	0.19	0.07, 0.51	0.001**
**rCBF ratio, %**	319	129	0.44	0.20, 0.96	0.040*

**Note:** ^1^OR = Odds Ratio, CI = Confidence Interval. ^2^*p<0.05; **p<0.01; ***p<0.001.

## Data Availability

All data generated or analyzed during this study can be obtained from the corresponding author upon reasonable request.

## LIST OF ABBREVIATIONS

AIC: Akaike Information Criterion
AIS: Athens Insomnia Scale
BMI: Body Mass Index
CBF: Cerebral Blood Flow
CBT-I: Cognitive Behavioral Therapy for Insomnia
CBV: Cerebral Blood Volume
CI: Confidence Interval
CNS: Central Nervous System
DSA: Digital Subtraction Angiography
DSC-MRI: Dynamic Susceptibility Contrast-Magnetic Resonance Imaging
EDAS: Encephaloduroarteriosynangiosis
EEG: Electroencephalography
ICA: Internal Carotid Artery
MCA: Middle Cerebral Artery
MMD: Moyamoya Disease
MMS: Moyamoya Syndrome
MoCA: Montreal Cognitive Assessment
mRS: Modified Rankin Scale
OR: Odds Ratio
OSA: Obstructive Sleep Apnea
PCA: Posterior Cerebral Artery
PSG: Polysomnography
PSQI: Pittsburgh Sleep Quality Index
rCBF: relative Cerebral Blood Flow
rCBV: relative Cerebral Blood Volume
RCS: Restricted Cubic Spline
REM: Rapid Eye Movement
ROI: Region of Interest
SAS: Self-rating Anxiety Scale
SDS: Self-rating Depression Scale
SWS: Slow Wave Sleep
TIA: Transient Ischemic Attack
TTP: Time-to-Peak

## STANDARDS OF REPORTING

STROBE guidelines were followed.

## References

[1] Suzuki J., Takaku A. (1969). Cerebrovascular “moyamoya” disease. Disease showing abnormal net-like vessels in base of brain.. Arch. Neurol..

[2] Ihara M., Yamamoto Y., Hattori Y., Liu W., Kobayashi H., Ishiyama H., Yoshimoto T., Miyawaki S., Clausen T., Bang O.Y., Steinberg G.K., Tournier-Lasserve E., Koizumi A. (2022). Moyamoya disease: Diagnosis and interventions.. Lancet Neurol..

[3] Sateia M.J. (2014). International classification of sleep disorders-third edition: Highlights and modifications.. Chest.

[4] Hermann D.M., Bassetti C.L. (2009). Sleep-related breathing and sleep-wake disturbances in ischemic stroke.. Neurology.

[5] Somers V.K., White D.P., Amin R., Abraham W.T., Costa F., Culebras A., Daniels S., Floras J.S., Hunt C.E., Olson L.J., Pickering T.G., Russell R., Woo M., Young T. (2008). Sleep apnea and cardiovascular disease.. J. Am Coll. Cardiol..

[6] McDermott M., Brown D.L., Chervin R.D. (2018). Sleep disorders and the risk of stroke.. Expert Rev. Neurother..

[7] Khot S.P., Morgenstern L.B. (2019). Sleep and stroke.. Stroke.

[8] Fujimura M., Tominaga T., Kuroda S., Takahashi J.C., Endo H., Ogasawara K., Miyamoto S. (2022). 2021 Japanese Guidelines for the Management of Moyamoya Disease: Guidelines from the Research Committee on Moyamoya Disease and Japan Stroke Society.. Neurol. Med. Chir..

[9] Nasreddine Z.S., Phillips N.A., Bédirian V., Charbonneau S., Whitehead V., Collin I., Cummings J.L., Chertkow H. (2005). The montreal cognitive assessment, MoCA: A brief screening tool for mild cognitive impairment.. J. Am Geriatr. Soc..

[10] Bigi S., Fischer U., Wehrli E., Mattle H.P., Boltshauser E., Bürki S., Jeannet P.Y., Fluss J., Weber P., Nedeltchev K., El-Koussy M., Steinlin M., Arnold M. (2011). Acute ischemic stroke in children versus young adults.. Ann. Neurol..

[11] Han Q., Liu B., Lin S., Li J., Liang P., Fu S., Zheng G., Yang S., Li B., Yang Q. (2021). Pittsburgh sleep quality index score predicts all-cause mortality in chinese dialysis patients.. Int. Urol. Nephrol.

[12] Jokelainen J., Timonen M., Keinänen-Kiukaanniemi S., Härkönen P., Jurvelin H., Suija K. (2019). Validation of the zung self-rating depression scale (SDS) in older adults.. Scand. J. Prim. Health. Care.

[13] Samakouri M., Bouhos G., Kadoglou M., Giantzelidou A., Tsolaki K., Livaditis M. (2012). Standardization of the greek version of zung’s self-rating anxiety scale (SAS).. Psychiatriki.

[14] Okajima I., Miyamoto T., Ubara A., Omichi C., Matsuda A., Sumi Y., Matsuo M., Ito K., Kadotani H. (2020). Evaluation of severity levels of the athens insomnia scale based on the criterion of insomnia severity index.. Int. J. Environ. Res. Public. Health..

[15] Qiao P.G., Han C., Zuo Z.W., Wang Y.T., Pfeuffer J., Duan L., Qian T., Li G.J. (2017). Clinical assessment of cerebral hemodynamics in moyamoya disease via multiple inversion time arterial spin labeling and dynamic susceptibility contrast-magnetic resonance imaging: A comparative study.. J. Neuroradiol.

[16] Chen Y., Xu W., Guo X., Shi Z., Sun Z., Gao L., Jin F., Wang J., Chen W., Yang Y. (2016). CT perfusion assessment of Moyamoya syndrome before and after direct revascularization (superficial temporal artery to middle cerebral artery bypass).. Eur. Radiol..

[17] Alimoradi Z., Jafari E., Broström A., Ohayon M.M., Lin C.Y., Griffiths M.D., Blom K., Jernelöv S., Kaldo V., Pakpour A.H. (2022). Effects of cognitive behavioral therapy for insomnia (CBT-I) on quality of life: A systematic review and meta-analysis.. Sleep Med. Rev..

[18] Urbano F., Roux F., Schindler J., Mohsenin V. (2008). Impaired cerebral autoregulation in obstructive sleep apnea.. J. Appl. Physiol..

[19] Ng K.T., Lee Z.X., Ang E., Teoh W.Y., Wang C.Y. (2020). Association of obstructive sleep apnea and postoperative cardiac complications: A systematic review and meta-analysis with trial sequential analysis.. J. Clin. Anesth..

[20] Koren D., Taveras E.M. (2018). Association of sleep disturbances with obesity, insulin resistance and the metabolic syndrome.. Metabolism.

[21] Bubu O.M., Brannick M., Mortimer J., Umasabor-Bubu O., Sebastião Y.V., Wen Y., Schwartz S., Borenstein A.R., Wu Y., Morgan D., Anderson W.M. (2017). Sleep, cognitive impairment, and Alzheimer’s disease: A systematic review and meta-analysis.. Sleep.

[22] Su X., Wang D.X. (2018). Improve postoperative sleep.. Curr. Opin. Anaesthesiol.

[23] Mansukhani M.P., Bellolio M.F., Kolla B.P., Enduri S., Somers V.K., Stead L.G. (2011). Worse outcome after stroke in patients with obstructive sleep apnea: An observational cohort study.. J. Stroke Cerebrovasc. Dis..

[24] Togao O., Mihara F., Yoshiura T., Tanaka A., Noguchi T., Kuwabara Y., Kaneko K., Matsushima T., Honda H. (2006). Cerebral hemodynamics in moyamoya disease: Correlation between perfusion-weighted MR imaging and cerebral angiography.. AJNR Am J. Neuroradiol..

[25] Javaheri S., Redline S. (2017). Insomnia and risk of cardiovascular disease.. Chest.

[26] Pengo M.F., Won C.H., Bourjeily G. (2018). Sleep in women across the life span.. Chest.

[27] Foley D., Ancoli-Israel S., Britz P., Walsh J. (2004). Sleep disturbances and chronic disease in older adults.. J. Psychosom. Res..

[28] Foley D.J., Monjan A.A., Brown S.L., Simonsick E.M., Wallace R.B., Blazer D.G. (1995). Sleep complaints among elderly persons: An epidemiologic study of three communities.. Sleep.

[29] Ohayon M.M., Carskadon M.A., Guilleminault C., Vitiello M.V. (2004). Meta-analysis of quantitative sleep parameters from childhood to old age in healthy individuals: Developing normative sleep values across the human lifespan.. Sleep.

[30] Rains J.C., Poceta J.S., Penzien D.B. (2008). Sleep and headaches.. Curr. Neurol. Neurosci. Rep..

[31] Colrain I.M., Nicholas C.L., Baker F.C. (2014). Alcohol and the sleeping brain.. Handb. Clin. Neurol..

[32] Ebrahim I.O., Shapiro C.M., Williams A.J., Fenwick P.B. (2013). Alcohol and sleep I: effects on normal sleep.. Alcohol Clin. Exp. Res..

[33] Chang W.P., Lin C.C. (2015). Use of opioid analgesics or sleeping medication and survival of cancer patients.. Eur. J. Oncol. Nurs..

[34] Morgenthaler T., Kramer M., Alessi C., Friedman L., Boehlecke B., Brown T., Coleman J., Kapur V., Lee-Chiong T., Owens J., Pancer J., Swick T. (2006). Practice parameters for the psychological and behavioral treatment of insomnia: An update. An american academy of sleep medicine report.. Sleep.

[35] Baglioni C., Spiegelhalder K., Lombardo C., Riemann D. (2010). Sleep and emotions: A focus on insomnia.. Sleep Med. Rev..

[36] Jacobsen P.B., Jim H.S. (2008). Psychosocial interventions for anxiety and depression in adult cancer patients: Achievements and challenges.. CA Cancer J. Clin..

[37] Miyamoto S., Kikuchi H., Karasawa J., Nagata I., Ikota T., Takeuchi S. (1984). Study of the posterior circulation in moyamoya disease.. J. Neurosurg..

[38] Nishimoto A., Takeuchi S. (1968). Abnormal cerebrovascular network related to the internal cartoid arteries.. J. Neurosurg..

[39] Yamada I., Himeno Y., Nagaoka T., Akimoto H., Matsushima Y., Kuroiwa T., Shibuya H. (1999). Moyamoya disease: Evaluation with diffusion-weighted and perfusion echo-planar MR imaging.. Radiology.

[40] Bålfors E.M., Franklin K.A. (1994). Impairment of cerebral perfusion during obstructive sleep apneas.. Am. J. Respir. Crit. Care Med..

[41] Kamdar B.B., Needham D.M., Collop N.A. (2012). Sleep deprivation in critical illness: Its role in physical and psychological recovery.. J. Intensive Care Med..

[42] Smith M.T., Haythornthwaite J.A. (2004). How do sleep disturbance and chronic pain inter-relate? Insights from the longitudinal and cognitive-behavioral clinical trials literature.. Sleep Med. Rev..

[43] Krieger J., Vitiello M.V. (1997). Sleep medicine reviews.. Sleep Med. Rev..

[44] Hasan F., Gordon C., Wu D., Huang H.C., Yuliana L.T., Susatia B., Marta O.F.D., Chiu H.Y. (2021). Dynamic prevalence of sleep disorders following stroke or transient ischemic attack.. Stroke.

[45] Culebras A. (2004). Cerebrovascular disease and sleep.. Curr. Neurol. Neurosci. Rep..

